# Interleukin-15 in cancer immunotherapy: IL-15 receptor complex versus soluble IL-15 in a cancer cell-delivered murine leukemia model

**DOI:** 10.1186/s40425-019-0777-8

**Published:** 2019-12-19

**Authors:** Alexandra Berger, Sarah J. Colpitts, Melanie S. S. Seabrook, Caren L. Furlonger, Maura B. Bendix, Joshua M. Moreau, William M. McKillop, Jeffrey A. Medin, Christopher J. Paige

**Affiliations:** 1 Princess Margaret Cancer Centre, University Health Network, 610 University Avenue, Room 8-105, Toronto, Ontario M5G 2M9 Canada; 2 Department of Immunology, University of Toronto, Toronto, Canada; 3 Department of Dermatology, University of California San Francisco, San Francisco, USA; 4 Departments of Pediatrics and Biochemistry, Medical College of Wisconsin, Milwaukee, USA; 5 Department of Medical Biophysics, University of Toronto, Toronto, Canada

**Keywords:** Leukemia, Interleukin-15, Cancer immunotherapy, T-cells, NK-cells

## Abstract

Cytokines of the common γ-chain receptor family such as IL-15 are vital with respect to activating immune cells, sustaining healthy immune functions, and augmenting the anti-tumor activity of effector cells, making them ideal candidates for cancer immunotherapy. IL-15, either in its soluble form (IL-15sol) or complexed with IL-15Rα (IL-15Rc), has been shown to exhibit potent anti-tumor activities in various experimental cancer studies. Here we describe the impact of intraperitoneal IL-15 in a cancer cell-delivered IL-15 immunotherapy approach using the 70Z/3-L leukemia mouse model. Whereas both forms of IL-15 led to significantly improved survival rates compared to the parent cell line, there were striking differences in the extent of the improved survival: mice receiving cancer cells secreting IL-15sol showed significantly longer survival and protective long-term immunity compared to those producing IL-15Rc. Interestingly, injection of leukemia cells secreting IL-15sol lead to heightened expansion of CD4^+^ and CD8^+^ T-cell populations in the peritoneum compared to IL-15Rc. Cell-secreted IL-15Rc resulted in an influx and/or expansion of NK1.1^+^ cells in the peritoneum which was much less pronounced in the IL-15sol model. Furthermore, IL-15Rc but not IL-15sol lead to T-cell exhaustion and disease progression. To our knowledge, this is the first study detailing a significantly different biological effect of cell-delivered IL-15sol versus IL-15Rc in a mouse cancer immunotherapy study.

## Background

Cancer immunotherapy endeavors to stimulate the immune system in order to recognize, reject, and destroy tumor cells. A number of molecules have been investigated as potential immunomodulators in cancer immunotherapy and some have shown promising results in triggering immune responses against tumor antigens, thereby improving patient survival. Over the past two decades a growing interest in harnessing the immune system to eliminate cancer cells has been accompanied by efforts to better characterize the complex signaling networks behind cytokines and chemokines to develop new cancer treatments. Cytokines have the ability to directly stimulate immune effector cells and activate cytotoxic cells. Numerous animal tumor models have demonstrated broad anti-tumor activity for various cytokines, including GM-CSF, IL-2, IL-7, IL-12, IL-15, IL-18 and IL-21 [1, 2]. Previously we demonstrated that IL-12 is an ideal candidate for leukemia immunotherapy [3, 4] and a phase I clinical trial is currently testing this approach in leukemia patients. In this study we have investigated the potential of interleukin-15 (IL-15) as an immunomodulator in an intraperitoneal cell-delivered leukemia mouse model.

IL-15 is a proinflammatory cytokine, important for the differentiation and proliferation of T-cells, NK/T-cells, and the development of dendritic cells [5, 6]. IL-15 occurs in two forms: soluble IL-15 (IL-15sol), and complexed with its proprietary receptor IL-15Rα, forming the IL-15 receptor complex (IL-15Rc) [7]. IL-15Rc is trans-presented to neighboring cells expressing IL-15Rβ/γ, exerting enhanced bioactivity compared to IL-15sol alone. This mechanism of trans-presentation represents a precise delivery of the IL-15 stimulus, confined to selective microenvironments [8–14] and IL-15 trans-presentation seems to be the physiologically relevant IL-15 signal [15].

In this study we compared the effects of IL-15sol vs IL-15Rc in their role as immunomodulators in the 70Z/3-L leukemia mouse model in order to evaluate the potential of IL-15 as an immunotherapeutic. We evaluated whether leukemia cells engineered to secrete either IL-15sol or IL-15Rc would elicit an anti-leukemia immune response. We show that both forms of IL-15 led to significantly improved survival compared to the parent line. However, there were striking differences in the extent of that improved survival. Mice receiving cancer cells secreting IL-15sol showed near 100% survival for up to 250 days, whereas mice receiving cancer cells secreting IL-15Rc started to perish around day 50 post-injection, with few survivors left on day 250.

Interestingly, cell-secreted IL-15sol and IL-15Rc activated different cell types in the peritoneum, leading to different immune responses. While IL-15sol preferentially expanded CD8^+^ and especially CD4^+^ T-cells in the peritoneum, injection of IL-15Rc-secreting leukemia cells was followed by an influx and/or expansion of NK1.1^+^ cells in the peritoneum. Furthermore, IL-15Rc lead to T-cell exhaustion and disease progression.

To our knowledge, this is the first study detailing a significantly different biological effect of cell-delivered IL-15sol versus IL-15Rc in a mouse cancer immunotherapy study, suggesting a different mechanism of action of the two IL-15 forms. We hypothesize that IL-15 in its two forms may act as a switch, orchestrating the balance of the innate and adaptive immune system, resulting in a fine-tuned immune response. Further comprehension of the dynamic interplay of IL-15sol and IL-15Rc may be of utmost importance in our understanding of immune responses, cancer, and autoimmunity. 

## Materials and methods

### Animals

Female B6D2F1 mice, 8–12 weeks old, were purchased from the Ontario Cancer Institute and kept under sterile conditions in a specific pathogen-free animal facility. 10–16 week old mice were used in all experiments. At the end of any experiment (typically 100 days), animals were terminated by CO_2_ asphyxiation/cervical dislocation. All experimental procedures were approved by the Animal Care Committee of the Ontario Cancer Institute.

### Leukemia cell line

70Z/3-L leukemia cells, derived from B6D2F1 mice, were maintained in complete Opti-MEM: Opti-MEM (Gibco, USA) with 5% heat-inactivated fetal calf serum (FCS) (Gibco, USA), 1xpenicillin/streptomycin (Wisent, Canada), and 5.5 × 10^−5^M β-mercaptoethanol, in a humidified atmosphere at 37 °C and 5%CO^2^ [3, 16].

### Construction of lentivirus constructs LV15Rc and LV15sol

**Fig. 1:**
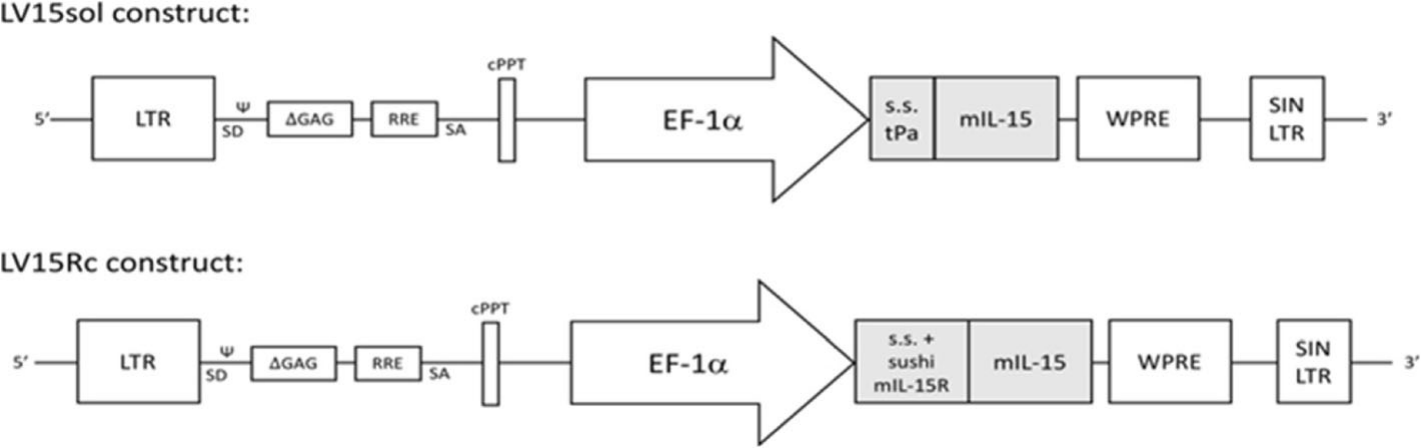
Construction of lentivirus constructs LV15sol and LV15Rc. Diagram of the lentiviruses (LVs) pDy.tpa-mIL15 (LV15sol) and pDY.mIL-15Rc (LV15Rc). Both LVs contain LTR, HIV long terminal repeat; Ψ, human immunodeficiency virus packaging sequence; SD, 5′ splice donor; ΔGAG, truncated group antigen sequence; RRE, Rev. response element; SA, 3′ splice acceptor; cPPT, central polypurine tract; EF-1α, elongation factor-1 alpha promoter; WPRE, woodchuck hepatitis virus post-transcriptional regulatory element; SIN/LTR, self-inactivating HIV long terminal repeat. LV15sol: The signal sequence (s.s.) and pro-peptide of tissue plasminogen activator (tPA) (amino acids 1–35 as predicted by Uniprot bioinformatic analyses) replaced the endogenous signal sequence and pro-peptide (amino acids 1–48 as predicted by Uniprot bioinformatic analyses) of mouse IL-15. A DNA cassette comprising a Kozak consensus sequence and this IL-15sol cDNA was synthesized by Genscript (Piscataway, USA) and subcloned into the lentiviral backbone pDY.cPPT-EF1α.WPRE downstream of the EF-1a promoter. LV15Rc: The first 98 amino acids of mouse IL-15Rα, including its signal peptide and sushi domain as identified by Prosite bioinformatic analysis (SIB Swiss Institute of Bioinformatics), were fused to mouse IL-15 amino acids 49–162 (signal and pro-peptide removed by Uniprot bioinformatic analyses) by a linker (SGGSGGGGSGGGSGGGGSLQ). A DNA cassette comprising a Kozak consensus sequence upstream of this IL-15Rc cDNA was synthesized by Genscript (Piscataway, USA) and subcloned into the 3′SIN, HIV-1-based, lentiviral backbone pDY.cPPT-EF1α WPRE, downstream of the EF-1α promoter. Both vectors were verified by restriction enzyme digestion and DNA sequencing. Lentiviral particles were produced at the University Health Network Vector Production Facility

We constructed two novel lentiviruses (LVs) (Fig. 1). LV15sol contains the signal sequence from tissue plasminogen activator, a commonly used signal sequence (s.s.) that designates the fused protein for secretion, fused to the cDNA of mouse IL-15. LV15Rc contains a partial cDNA of mouse IL-15Rα including its signal and pro-peptides as well as the sushi domain required for binding to IL-15 [17] linked to the mature processed mouse IL-15 cDNA. With human cDNA, this complex has been reported to display increased stability, secretion, and bioactivity in comparison to IL-15 alone [18].

### Viral transduction and IL-15 ELISAs

The parent leukemia cell line was transduced with an approximate multiplicity of infection of 10. After 3 washes cells were seeded into Terasaki plates at a density of 0.3 cells/well to ensure the presence of single-cell wells. Cells were expanded and clones were quantitated for the production of IL-15 at 10^6^ cells/ml/hour. The following ELISA kits were used: mouse IL-15 DuoSet ELISA for IL-15sol (DY447, R&D Systems, USA) and mouse IL-15/IL-15R complex ELISA for IL-15Rc (88–7215, Invitrogen/Thermo Fisher, USA).

### In vivo challenge

70Z/3-L lines were expanded in complete Opti-MEM, washed twice and resuspended at a density of 5 × 10^6^ cells/ml. Each B6D2F1 mouse received 10^6^ cells in a volume of 200 μl PBS injected into the left abdominal cavity using a 1 ml syringe and 26-gauge needle. Mice were then monitored for onset of sickness. The control group usually showed first signs of sickness around day 10. The end point of most experiments was set to day 100.

To evaluate protective immunity, surviving mice were re-challenged with 10^6^ cells of the parent strain after 100 days of the initial challenge.

### Depletion experiments

B6D2F1mice were depleted of T-cell subsets as well as NK-cells using specific depletion antibodies as previously described [3]. Depletion antibodies were injected ip at a dose of 0.5 mg antibody/mouse on days − 2, 3, 6, 10 and 13, and after that once weekly for another month. Cells were injected on “day 0”. Prior to depletion experiments, the efficacy of depletion antibodies was demonstrated in vivo by flow cytometry (data not shown).

### Generation of GFP^+^ leukemia cells

70Z/3-L cells were co-cultured with the GFP retroviral packaging line GP + E (ATCC, USA) to render them GFP^+^. After 48 h of co-culture, suspension 70Z/3-L leukemia cells were removed from the adherent packaging line, passed multiple times to ensure no cells of the packaging line were carried over, and expanded. GFP^+^ 70Z/3-L cells were sorted and the top 10% of GFP^+^ expressing cells were collected. GFP expression was stable, as revealed by repeated flow analysis. 10^6^ GFP^+^ leukemia cells were injected ip into mice to monitor cell expansion in vivo. GFP^+^ clones yielded similar survival compared to their non-GFP counterparts (data not shown).

### Flow cytometry analysis of the cell populations in the peritoneum

Cells were harvested from the peritoneum by peritoneal lavage with PBS containing 1%FCS. Peritoneal cells were washed, counted, and stained for 30 min with specific antibodies: CD4 (RM4–5; BioLegend), CD8 (53–6.7; BD Biosciences), NK1.1 (PK136; BioLegend), CD44 (IM7; BD Biosciences), CD62L (MEL-14; eBioscience), CD25 (PC61; BioLegend), FOXP3 (MF-14; BioLegend), PD-1 (29F.1A12; BioLegend). For GrzB staining cells were first stained with antibodies directed against antigens on the cell surface, then fixed overnight using the Foxp3 Fixation/Permeabilization Concentrate/Diluent kit (eBioscience, USA). The next morning, cells were permeabilized and stained with GzmB-FITC (GB11; BioLegend) for 45 min. Flow cytometry was performed using the FortessaX20 (Becton Dickinson). Analysis was done using FlowJo software (TreeStar).

## Results

### Generation of IL-15 secreting leukemia cells by lentiviral transduction

**Fig. 2:**
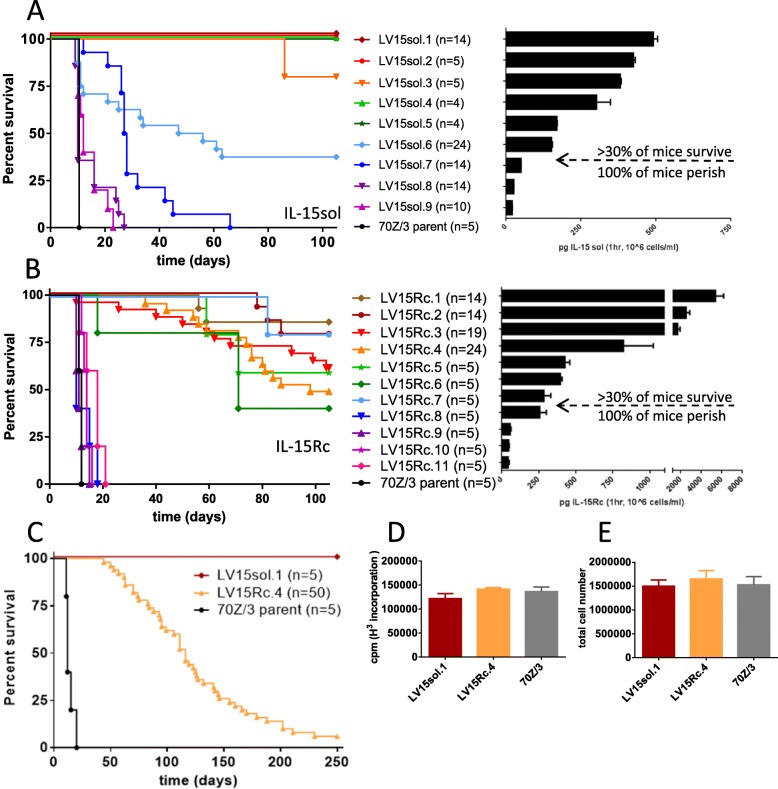
Increasing levels of leukemia cell-mediated IL-15 positively correlates with improved survival. **a** IL-15 levels of LV15sol clones (for both (A) and (B) secretion levels are mean + SEM calculated from 2 to 4 individual ELISAs with duplicate wells). **b** IL-15 levels of LV15Rc clones; Correlation of survival with IL-15 output: **a** LV15sol clones; (*p* <  0.005, LV15sol.1–.7 vs 70Z/3-L, Log-rank, Mantel-Cox test); **b** LV15Rc clones; (*p* <  0.0001 for LV15Rc.1–.4, *p* <  0.003 for LV15Rc.5–.7 vs 70Z/3-L, Log-rank, Mantel-Cox test); Mice were injected ip with 10^6^ cells of either the parent line, or one of the transduced clones and monitored for onset of disease. Based on the secretion levels of IL-15 a theoretical threshold was established (arrow indicates the threshold), below which the protective effect of IL-15 was not observed. Multiple experiments were pooled for survival curves for some clones (n numbers are indicated in brackets). **c** Side by side comparison of the survival rates of the two IL-15 models using clones LV15sol.1 and LV15Rc.4 in an extended 250-day experiment. *P*-values: *p* <  0.001 for both IL-15 groups vs 70Z/3-L controls; *p* = 0.0003 LV-15Rc vs LV-15sol (Log-rank, Mantel-Cox test); **d** H^3^-Thymidin incorporation and **e** total live cell counts demonstrate that the representative LV15Rc and LV15sol clones used throughout this study grow at a similar rate as the 70Z/3-L parent strain. Results are mean + SEM calculated from 2 to 4 individual experiments with triplicate wells

Transduced 70Z/3-L clones were evaluated for their production of IL-15sol and IL-15Rc by ELISA. IL-15sol was expressed at levels ranging from approximately 40–500 pg/ml/hr./10^6^cells among various clones (Fig. 2a) which remained stable over time as determined by repeated testing of the clones (data not shown). The range of secretion of IL-15Rc was approximately 100–5000 pg/ml/hr./10^6^cells among clones (Fig. 2b) which also remained stable over time (data not shown).

For comparison purposes we picked a representative clone of each model that secreted similar levels of IL-15: LV15sol.1 secretes 500 ± 50 pg/ml/hr./10^6^cells of IL-15sol; LV15Rc.4 secretes 750 ± 300 pg/ml/hr./10^6^cells of IL-15Rc. Therefore, IL-15 levels secreted by LV15sol.1 and LV15Rc.4 are not statistically different (*p* = 0.3353, unpaired t-test). Furthermore, both clones show similar growth rates as determined by H^3^-Thymidine incorporation (Fig. 2d) and cell counts (Fig. 2e). LV15sol.1 was chosen because we wanted the clone that secreted the highest levels of IL-15sol as IL-15sol levels tend to be low overall. LV15Rc.4 was picked because it secretes similar levels to IL-15sol.1 and showed a similar growth rate.

### Leukemia cell clones producing either LV15sol or LV15Rc prolong survival

In order to determine whether the secretion of either IL-15sol or IL-15Rc by the transduced leukemia cells would elicit a protective immune response in the host, a number of clones spanning a range of IL-15 secretion levels were injected into the peritoneal cavity of B6D2F1 mice (10^6^cells/mouse).

We saw a clear trend of improved survival with higher concentrations of IL-15. Clones producing less than 200 pg/ml/hr./10^6^cells of IL-15sol failed to elicit a protective immune response and mice perished around the same time as the control group (Fig. 2a). Clones secreting more than 200 pg/ml/hr./10^6^cells of IL-15sol elicited a protective immune response which led to long-term survival of almost 100% of mice on day 100 (Fig. 2a).

Similarly, clones producing less than 250 pg/ml/hr./10^6^cells of IL-15Rc failed to elicit a protective immune response (Fig. 2b). Clones secreting more than 250 pg/ml/hr./10^6^cells of IL-15Rc elicited an immune response leading to partial protection and a proportion of mice survived up to 100 days (Fig. 2b). However, mice started dying after 50 days post-injection, suggesting incomplete leukemia cell clearance.

To better understand the survival of IL-15Rc leukemia cell-bearing mice a larger experiment with 50 mice in the Rc-cohort was launched using the clones LV15sol.1 and LV15Rc.4, which secrete similar levels of IL-15 as determined by ELISA and shown in Fig. 2. This study demonstrated that over the course of 250 days almost all mice in the Rc cohort perished (Fig. 2c). The experiment was terminated with only 3 mice alive in the IL-15Rc group (3/50 = 6%). Conversely, all mice that had received cancer cells secreting IL-15sol survived the 250 days (Fig. 2c), suggesting a different mechanism of action for IL-15Rc and IL-15sol. It should be noted that clones secreting the highest levels of IL-15Rc fared no better than mid-range level clones, indicating that the IL-15 threshold has been reached by mid-level.

To test for the presence of residual tumor cells in surviving mice on day 100, splenocytes and peritoneal cells were cultured without growth factors, and monitored for the growth of 70Z/3-L cells. Cultures generated from mice injected with IL-15sol-secreting leukemia cells were free of leukemia cells (*n* = 10 mice). However, 100% of peritoneal cultures and 90% of splenic cultures harvested from mice injected with IL-15Rc-secreting cancer cells were confluent with 70Z/3-L cancer cells after 2–3 weeks (n = 10 mice). These IL-15Rc clones secreted similar levels of IL-15Rc as prior to injection (data not shown). The cells were further re-injected into naïve mice to test whether 100 days in vivo had changed their properties. However, they yielded similar survival patterns to the original clones (data not shown).

### Leukemia cell-mediated IL-15sol therapy leads to long-term protective immunity against 70Z3-L leukemia while IL-15Rc therapy does not

**Fig. 3:**
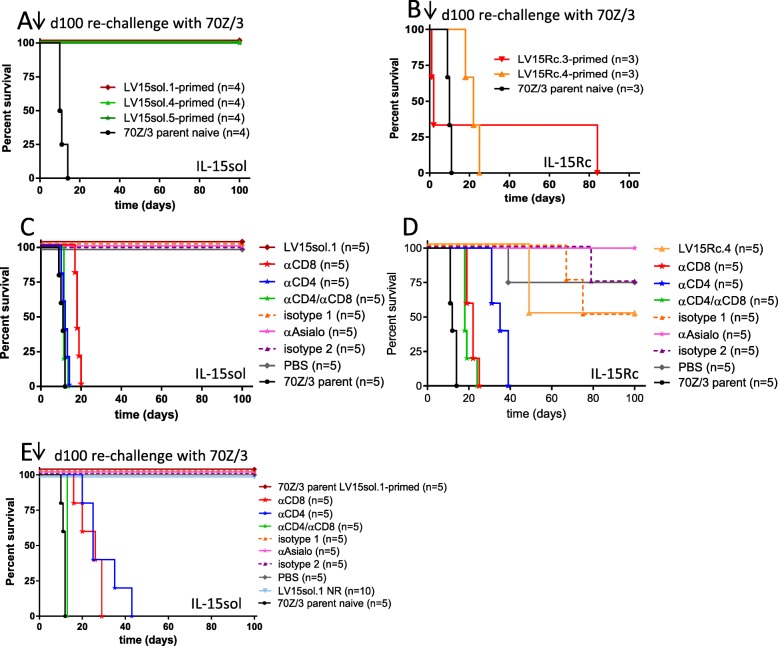
Leukemia cell-mediated IL-15 therapy leads to long-term immunity and protection against 70Z/3-L for IL-15sol but not IL-15Rc. **a, b** Mice were initially injected with 10^6^ cells of either **a** LV15sol clones, or **b** LV15Rc clones. After 100 days surviving mice were re-challenged with 10^6^ 70Z/3-L cells to test whether immunity was established. A naïve control group received 10^6^ 70Z/3-L cells to control for the efficiency to cause leukemia. **a** *p* = 0.0062 for all 3 surviving groups vs 70Z/3-L controls; **b** *p* = 0.0246 (LV15Rc.3-primed), *p* = 0.9876 (LV15Rc.4-primed) vs 70Z/3-L controls; **c-e** Both T-cell subsets are required for leukemia cell-mediated IL-15 therapy**.** Mice were depleted of certain cell populations prior to challenging them with either **c** 10^6^ LV15sol.1 cells; or **d** 10^6^ LV15Rc.4 cells. In both instances, CD4^+^ and CD8^+^ T-cell subsets were required. **e** In order to establish whether the same T-cell populations are necessary in the secondary challenge, we immunized 55 mice with the IL-15sol clone LV15sol.1. After 100 days (all 55 mice survived) they were depleted of various cell populations and then re-challenged with the parent strain. Again, both T-cell subsets were required. In all instances, a control group (naïve mice) received 10^6^ 70Z/3-L cells to control for their efficiency to cause leukemia. In the re-challenge experiment one group of not re-challenged (NR) mice was included. Depletion of NK-cells did not have an effect in any of the experiments

One hundred days after the initial ip injection of 10^6^ IL-15-secreting leukemia cells, surviving mice were re-challenged with 10^6^ cells of the parent cell line in order to test whether long-lasting, IL-15-independent, immunity had been established. To test for the efficacy of 70Z/3-L to induce leukemia, a naïve control group was injected with the parent line. All mice surviving the initial injection of cells secreting IL-15sol survived the re-challenge for an additional 100 days (Fig. 3a), suggesting that IL-15sol bestows long-lasting protective immunity. While cell-mediated IL-15sol therapy leads to immunity, IL-15Rc therapy does not (Fig. 3b).

### Both T-cell subsets are required for IL-15-mediated rejection of leukemia cells

Antibody-depletion is commonly used to eliminate various lymphocyte subsets in vivo. It is a useful tool to analyze the roles of different cellular subsets in immune responses and immunological diseases. Both CD4^+^ and CD8^+^ T-cells were essential to establish protective immunity in the IL-15sol (Fig. 3c) as well as the IL-15Rc (Fig. 3d) model, as depleting either T-cell subset ablated the protection. There were no fatalities in any of the isotype or PBS control groups in the IL-15sol model (Fig. 3c). Due to the poor long-term survival of mice receiving IL-15Rc-secreting leukemia cells we saw fatalities in the isotype and PBS groups (Fig. 3d), reflecting the results in Fig. 2c. Depleting NK-cells using the depletion antibody Anti-Asialo GM1 did not lead to deaths in either IL-15 model.

CD4^+^ and CD8^+^ T-cell populations were also necessary for survival after re-challenge and thus IL-15sol-induced long-term protective immunity (Fig. 3e). Due to the poor long-term survival of mice receiving IL-15Rc-secreting leukemia cells a secondary immune challenge was not performed.

### Cell-mediated IL-15sol and IL-15Rc induced different cytokine/chemokine profiles

The fact that IL-15sol and IL-15Rc-mediated cancer cell therapy revealed dramatically different survival rates prompted us to search for differences between IL-15sol- and IL-15Rc-mediated responses. Since cytokine treatment induces the secretion of other factors, we measured the levels of various cytokines/chemokines in the serum of mice that received IL-15sol vs IL-15Rc-secreting cancer cells. Blood was taken from a group of mice before as well as post-injection on days 8, 18, 30 and 40. 10 μl of serum was diluted with 40 μl of PBS and cytokine levels were measured using a 31-plex analysis (EveTechnologies, Clagary, Canada).

**Fig. 4:**
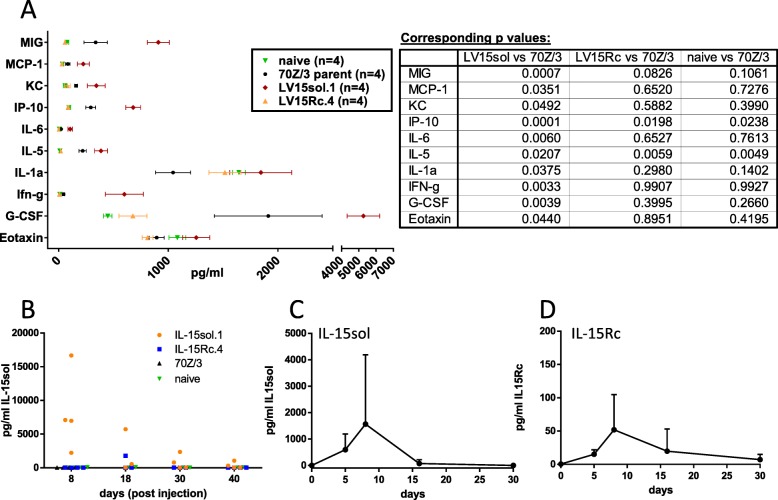
Serum cytokine profile of mice injected with IL-15-secreting leukemia cells. **a** Serum was taken eight days post ip-administration of leukemia cells. A control group of naïve mice was included. Results shown are mean + SEM calculated from 4 individual mice/group from one representative experiment performed at least twice. Corresponding *p* values can be found next to the graph. **b** Only IL-15sol could be detected in mouse serum using the 31-plex analysis (EveTechnologies, Calgary). We repeated the analysis of IL-15 in mouse serum using our ELISA systems to detect IL-15sol **c** as well as IL-15Rc **d**. Both **c** and **d** show a time course where mice were bled prior to injection and then on days 5, 8, 16 and 30 post-injection of IL-15 secreting leukemia cells

The analysis revealed different serum cytokine/chemokine profiles in host mice injected with leukemia cells secreting IL-15sol vs IL-15Rc. Serum of mice that had received cancer cells secreting IL-15sol showed significantly elevated levels for Eotaxin, G-CSF, IFN-γ, IL-1α, IL-5, IL-6, IP-10, KC, MCP-1 and MIG (Fig. 4a). Interestingly, serum from mice injected with IL-15Rc clones resembled serum cytokine levels of naïve mice, and serum taken from 70Z/3-L-injected mice had slightly elevated serum cytokine/chemokine levels (Fig. 4a). Fig. 4a shows the result of day-8 serum, which represented the peak of the response. To confirm this observation was not unique to the clones used in this experiment, additional clones were tested and showed similar results (data not shown).

IL-15 is also included in the 31-plex analysis. However, in mouse no cross-reactivity can be observed between IL-15sol and IL-15Rc [18]. Hence only IL-15sol was detected in mouse serum in the 31-plex analysis, with large variations between mice primed with IL-15sol.1 (Fig. 4b). Other clones of IL-15sol yielded similar results (data not shown). To test whether we could detect both forms of IL-15 using our ELISAs we performed them side by side using the same clones shown in Fig. 4b. Similar to Eve Technologies we could detect IL-15sol at varying levels in serum, peaking around day 7/8 (Fig. 4c). IL-15Rc serum levels were about 10-fold lower (Fig. 4d). In day-7 peritoneal fluids, both forms of IL-15 were readily detectable (7058.5 ± 5411.5 pg/ml IL-15Rc; 77,438 ± 4761.7 pg/ml IL-15sol; *n* = 2). In mice of the IL-15Rc model that showed signs of disease progression 50+ days post-injection we measured 156 ± 2.8 pg/ml IL-15Rc in serum and 1725.5 ± 219.9 pg/ml IL-15Rc in peritoneal fluid (*n* = 2).

### Kinetics of in vivo leukemia cell killing in IL-15sol vs IL-15Rc cell-mediated immunity

**Fig. 5:**
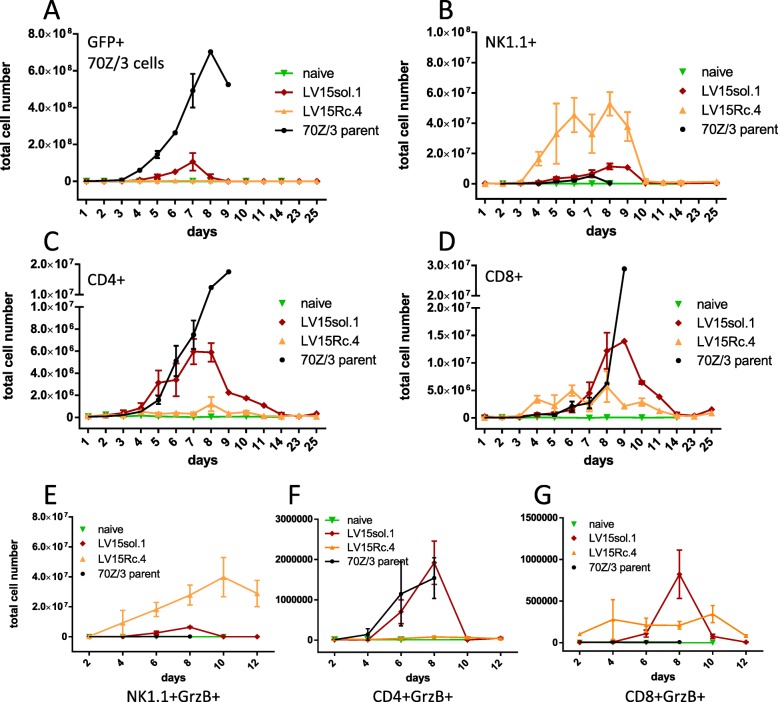
Kinetics of expansion of (A) leukemia, (B) NK1.1^+^, (C) CD4^+^, (D) CD8^+^, (E) NK1.1^+^GrzB^+^, (F) CD4^+^GrzB^+^, and (G) CD8^+^GrzB^+^ cells in the peritoneum of mice injected with IL-15 secreting leukemia cells. Every graph shows a time course, and every time point has been obtained by sacrificing 3–6 mice/group. Numbers shown are total cell numbers calculated based on the absolute cell numbers obtained by peritoneal lavage. Naïve mice were included to obtain baseline levels and were included on the graph on the day they were analyzed. Experiments have been performed three times with similar results, and were pooled. For statistical analysis peak values on day 8 were compared within groups using 1-way ANOVA (see Table 2 for statistical analysis). **a** GFP^+^70Z/3-L parent strain expands significantly more in vivo than LV15Rc or LV-15sol secreting line. **b** NK1.1^+^ cells expand significantly more in mice injected with LV15Rc.4 compared to all other groups. **c** CD4^+^ cells expand significantly more in mice injected with LV15sol.1 compared to all other groups. **d** CD8^+^ cells expand significantly more in mice injected with LV15sol.1 vs LV15Rc.4. **e** The increase of NK1.1^+^GrzB^+^ cells in the peritoneum of mice injected with LV15Rc.4 was significantly higher compared to LV15sol.1 (*p* = 0.0147, day 8); **f** The total numbers of CD4^+^GrzB^+^ cells in the peritoneum of mice injected with LV15sol.1 were significantly higher compared to LV15Rc.4 (*p* = 0.0070, day 8). **g** The total numbers of CD8^+^GrzB^+^ cells in the peritoneum of mice injected with LV15sol.1 were significantly higher compared to LV15Rc.4 (*p* = 0.0171, day 8)

The differences in survival together with our cytokine results suggest a different mechanism of action between the immune responses initiated by IL-15sol vs IL-15Rc. We next turned to flow cytometry to investigate the fate of the leukemia cells in the peritoneum. GFP experiments revealed that IL-15sol-secreting leukemia cells expanded in the host peritoneal cavity approximately 100-fold (from 10^6^ to about 10^8^), peaking around day 7, before they were eliminated by the immune system (Fig. 5a). Interestingly, cancer cells secreting IL-15Rc expanded only 2–5-fold in the host peritoneal cavity (Fig. 5a). Fig. 5a also depicts the parent cancer cell line which expanded to almost 10^9^ cells which ultimately led to the death of the host around day 10. The dramatic difference in cancer cell expansion and clearing between IL-15Rc and IL-15sol may be due to the activation of different cell types.

### IL-15Rc expands NK1.1^+^ cells, whereas IL-15sol expands CD4^+^ and CD8^+^ T-cells

Flow cytometry further revealed that IL-15sol lead to a heightened increase of both CD4^+^ (Fig. 5c) and CD8^+^ T-cell numbers (Fig. 5d) in the peritoneum compared to IL-15Rc, suggesting that IL-15sol is a better activator of T-cells. IL-15Rc on the other hand, is a potent activator of NK1.1^+^ cells, leading to a massive influx and/or expansion of NK1.1^+^ cells in the peritoneal cavity, while NK1.1^+^ cells only increased marginally in the presence of IL-15sol-secreting cells (Fig. 5b). We next tested whether these IL-15Rc-induced NK1.1^+^ cells, stained positive for Granzyme-B (GrzB). Figure 5e shows an approximately 10-fold increase of GrzB^+^NK1.1^+^ cell numbers in the peritoneum of mice injected with IL-15Rc-secreting cancer cells compared to IL-15sol, naïve mice, and mice injected with the parent strain. On the contrary, we saw significantly higher numbers of GrzB^+^CD4^+^ cells (Fig. 5f) and GrzB^+^CD8^+^ cells (Fig. 5g) in the peritoneum of mice injected with leukemia cells secreting IL-15sol compared to IL-15Rc.

### 50+ days post-injection T-cells in IL-15Rc mice express the exhaustion marker PD-1

**Fig. 6:**
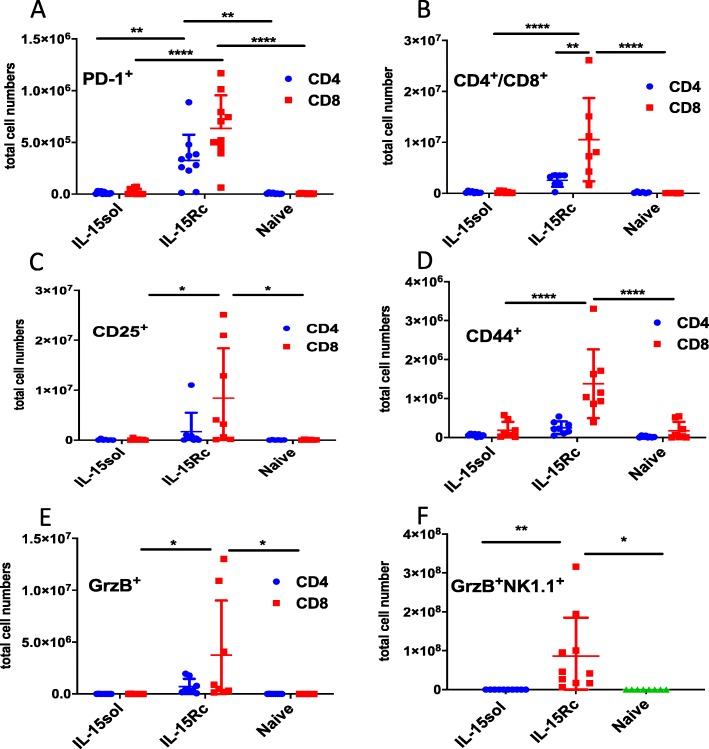
T-cells of mice injected with IL-15Rc secreting leukemia cells express significantly higher levels of exhaustion and activation markers 50+ days post-ip-injection. **a** PD-1; **b** CD4^+^/CD8^+^; **c** CD25^+^; **d** CD44^+^; **e** GrzB^+^ and **f** NK1.1^+^GrzB^+^ cells. Each data point represents 1 mouse. Numbers shown are total cell numbers calculated based on the absolute cell numbers obtained by peritoneal lavage. Naïve mice were included to obtain baseline levels and were included on the graph on the day they were analyzed. Experiments have been performed four times with 1–2 mice sacrificed per group, and results were pooled. For statistical analysis mean values were compared within groups using 2-way ANOVA with Tukey’s post-test. *P*-values are denoted with asterisks (* = *p* = < 0.05,** = *p* = < 0.01, *** = *p* = < 0.001, **** = *p* = < 0.0001)

Since mice injected with IL-15Rc-secreting tumor cells failed to develop long-term protective immunity, we wanted to test whether their T-cells were exhausted. We investigated whether PD-1 was upregulated on T-cells of IL-15Rc mice 50^+^ days post-injection. We found significantly more CD4^+^ and CD8^+^ T-cells expressing PD-1 in the peritoneal cavity of IL-15Rc mice compared to IL-15sol mice (Fig. 6a).

We further noticed that IL-15Rc mice have an increased total number of CD8^+^ T-cells 50^+^ days post-injection in comparison to IL-15sol mice (Fig. 6b). CD8^+^ T-lymphocytes in the peritoneum of IL-15Rc mice further showed an increase in activation markers such as CD25, CD44, and GrzB (Fig. 6c-e).

Since IL-15Rc triggered a large expansion of NK1.1^+^GrzB^+^ cells in the initial immune response which then quickly subsided (Fig. 5b), we investigated whether this subset also expanded again later in disease progression. IL-15Rc mice had significantly greater populations of NK1.1^+^GrzB^+^ cells 50^+^ days post-injection in comparison to IL-15sol mice (Fig. 6f).

## Discussion

We have previously shown for IL-12 that an intra-peritoneal (ip) cell-mediated delivery strategy embodies an effective form of leukemia immunotherapy which can circumvent toxicity issues often experienced when cytokines are delivered systemically [3]. In the present study we demonstrate that ip-administered IL-15-secreting leukemia cells can elicit a protective immune response in the 70Z/3-L mouse leukemia immunotherapy model.

In order to determine whether IL-15 would extend survival in the 70Z/3-L leukemia mouse model over the parent line we established stable 70Z/3-L clones that produce a range of IL-15. We found that IL-15sol clones produced lower levels of IL-15 than IL-15Rc clones, probably due to a shorter half-life. Above a threshold of about 200 pg/ml/hr./10^6^cells cell-secreted IL-15sol significantly improved the survival of mice compared to the parent cell line, leading to almost 100% survival of host mice up to 100 days (Fig. 2a). A similar trend of improved survival with higher secretion levels could be seen for IL-15Rc (Fig. 2b). However, although the highest IL-15Rc secreting clones produce about 10 times more IL-15 than the highest IL-15sol producing clone, the overall survival of mice receiving IL-15Rc-secreting leukemia cells was significantly shorter than that of host mice injected with IL-15sol leukemia cells (Fig. 2b). While in this study we only measure cytokine secretion levels we recognize that biological effects of cytokines can depend on binding affinity and half-life as well. For comparison purposes throughout this study, we chose clones LV15sol.1 and LV15Rc.4 which secrete similar levels of IL-15 as determined by ELISA (Fig. 2).

We further addressed the observed shortened survival of mice receiving IL-15Rc-secreting leukemia cells in vitro. Cultures generated from splenocytes and peritoneal cells of surviving mice that were injected with IL-15sol-secreting leukemia cells 100 days earlier, were free of leukemia cells, while cancer cells quickly overgrew cultures generated from mice injected with IL-15Rc-secreting leukemia cells. This confirms that residual leukemia cells only occur in host mice injected with the latter. Since the residual leukemia cells secreted similar levels of IL-15Rc as prior to injection we eliminate the loss of IL-15Rc as a reason as to why these leukemia cells evaded the immune system.

We further show by re-challenging with the parent cells that animals injected with IL-15sol-secreting leukemia cells mounted a successful long-lasting immune response, suggesting that IL-15sol not only cleared 70Z/3-L cells but also elicited long-term protective immunity (Fig. 3a). Mice of the IL-15Rc model lacked long-term protective immunity (Fig. 3b). Considering the issue of the residual leukemia cells in mice that had received IL-15Rc-secreting cells, the lack of protective immunity is not surprising. We most likely overburdened the system by re-challenging, which could explain why mice perished quickly after.

In order to find an explanation for the observed differences in survival and immunity, we examined the cytokine and chemokine profile in serum of mice injected with IL-15-secreting cancer cells, shedding light on the systemic nature of these molecules. Levels of IFN-γ, MIG, IP-10, IL-1α, IL-6, MCP-1 and G-CSF were significantly elevated in the serum of IL-15sol mice, whereas the IL-15Rc serum resembled naïve mice (Fig. 4a). IFN-γ is crucial when it comes to limiting cancer growth. It is produced predominantly by Th1 CD4^+^ and CD8^+^ cytotoxic T-cells once antigen-specific immunity develops. Being able to detect IFN-γ and its inducible proteins only in serum of mice injected with IL-15sol-secreting cells points to possible differences in T-cell activation.

In order to assess the cellular composition of the peritoneum of recipient mice, we turned to flow cytometry. We first addressed the fate of the leukemia cells in the peritoneum. Interestingly, the form of IL-15 mattered in the context of ip-expansion of leukemia cells. IL-15sol-secreting leukemia cells expanded about 100-fold compared to the initial number of injected cells before they were completely cleared by the immune system. IL-15Rc-secreting leukemia cells expanded only 2–5-fold, suggesting that IL-15Rc led to a quick but incomplete elimination of leukemia cells soon after injection (Fig. 5a). We postulate that this cell expansion explains the serum levels of IL-15. IL-15sol can be readily detected in day 8 serum (Fig. 4b,c), which is when the expansion of IL-15sol-secreting leukemia cells is at its peak. IL-15Rc-secreting leukemia cells barely expand in the primary challenge, which is probably why IL-15Rc levels are very low in serum (Fig. 4b,d). However, IL-15 levels are much higher in peritoneal fluid than serum and both forms of IL-15 are readily detectable in day-7 samples. Similarly, levels of IL-15Rc were high in serum as well as peritoneal fluid of mice of the IL-15Rc model at the time of disease progression 50+ days post-injection.

Most interestingly, leukemia cell-secreted IL-15sol and IL-15Rc affected different cell types in vivo (Fig. 5, Table 1). Both forms of IL-15 resulted in an increase of CD8^+^ T-cell proportions in the peritoneum, albeit to a different extent: 18.07 ± 1.9% CD8^+^ T-cells in the peritoneum of the IL-15sol model; 8.08 ± 1.87CD8^+^ T-cells in the IL-15Rc model; compared to < 1.1% in naïve mice and mice injected with the parent line. The proportion of CD4^+^ T-cells remained similar in hosts injected with IL-15Rc-secreting leukemia cells, naïve mice and mice injected with the parent line (under 2.5%), but was significantly increased to 9.72 ± 0.51% in the IL-15sol model, maybe suggesting the need for IL-15sol in the expansion of CD4^+^ helper T-cells, known to be vital for adaptive immune responses.

**Table 1:** Percentage of cell types on day 8 as presented in Fig. 5, calculated as mean ± SEM (*n* = 3) for all mouse groups: 70Z/3-L parent, naïve, LV15Rc.4, and LV15sol.1

Cell population	70Z/3 parent	naive	LV15Rc.4	LV15sol.1
GFP^+^ leukemia cells	84.84 ± 2.5%	n/a	6.19 ± 1.42	10.91 ± 1.7%
CD4^+^	1.44 ± 0.09%	1.74 ± 0.58%	2.33 ± 0.68%	9.72 ± 0.51
CD8^+^	0.47 ± 0.14%	1.11 ± 0.13	8.08 ± 1.87%	18.07 ± 1.9%
NK1.1^+^	4.3 ± 2.54%	2.57 ± 0.73	47.62 ± 4.96%	7.68 ± 0.57%
‘other’	8.96 ± 2.7%	94.57 ± 1.12	35.77 ± 5.86%	53.62 ± 1.85%

While leukemia cell-secreted IL-15sol was a more potent inducer of T-cells, IL-15Rc lead to an influx and/or expansion of NK1.1^+^ cells in the peritoneum. The proportion of NK1.1^+^ cells in the peritoneum of mice injected with IL-15Rc-secreting cancer cells reached an astounding 47.62 ± 4.96% compared to 7.68 ± 0.57% in the IL-15sol model and < 4.5% in naïve mice and mice injected with the parent line (Table 1). Statistical analysis for the cell numbers presented in Table 1 can be found in Table 2. Mice that had received LV15sol-expressing leukemia cells had significantly higher numbers of CD4^+^ cells compared to all other groups, whereas significantly elevated numbers of NK1.1^+^ cells could be found in mice that had received LV15Rc-expressing leukemia compared to all other groups (Table 2).

**Table 2:** Statistical significance of cell distribution between the mouse groups depicted in Table 1 (*p*-values as calculated by 1-way ANOVA followed by Tukey’s multiple comparison test)

	GFP^+^ 70Z/3	CD4^+^	CD8^+^	NK1.1^+^	‘other’
70Z/3 vs naive	n/a	0.9753	0.9856	0.9710	< 0.0001
70Z/3 vs LV15Rc.4	< 0.0001	0.6305	0.0159	< 0.0001	0.0023
70Z/3 vs LV15sol.1	< 0.0001	< 0.0001	< 0.0001	0.8314	< 0.0001
Naïve vs LV15Rc.4	n/a	0.8463	0.0251	< 0.0001	< 0.0001
Naïve vs LV15sol.1	n/a	< 0.0001	< 0.0001	0.5992	0.0001
LV15Rc.4 vs LV15sol.1	0.2633	< 0.0001	0.0033	< 0.0001	0.0247

We consistently saw increased numbers of Granzyme-B^+^ (GrzB^+^) cytotoxic T-cells in the IL-15sol model (Fig. 5f,g), suggesting killing predominately by IL-15sol-activated T-cells. In the peritoneum of mice injected with IL-15Rc-secreting cells we detected 10-fold elevated numbers of GrzB^+^NK1.1^+^ cells (Fig. 5e), consistent with the hypothesis of Granzyme-B-mediated killing of cancer cells by IL-15Rc-activated NK1.1^+^-cells. Although GrzB^+^NK1.1^+^ cells are activated and cytotoxic, they were not able to rescue the mice upon disease progression.

**Fig. 7:**
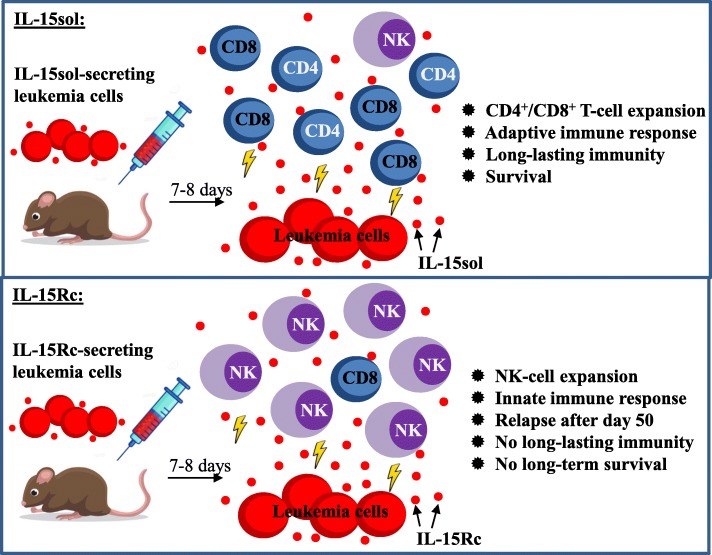
Summary of data of our IL-15 70Z/3 leukemia mouse model. Injection of IL-15sol-secreting leukemia cells leads to an intraperitoneal expansion of CD4+ and CD8+ T-cells within 7-8 days. The resulting adaptive immune response leads to long-lasting immunity and survival of the host. Injection of IL-15Rc-secreting leukemia cells leads to the massive intraperitoneal expansion of NK1.1+ cells within 7-8 days. The resulting innate-like immune response leads to the quick but incomplete killing of leukemia cells, which eventually results in a relapse, wide-spread disease, and thus the lack of long-lasting immunity and survival

Based on our results we hypothesize that IL-15sol secreted by the leukemia cells leads to immunity and long-term survival, because it results in an adaptive long-lasting immune response involving predominantly CD4^+^ and CD8^+^ T-cells (Fig. 7). IL-15Rc on the other hand leads to a disproportionate increase of NK-cells. Although these NK-cells kill the invading leukemia cells promptly, they seem to do so in an incomplete fashion, which leads to a relapse after day 50, eventually causing widespread disease (Fig. 7). This could also explain our earlier observation where ‘above threshold levels’ of IL-15sol lead to complete survival up to 250 days while IL-15Rc did not, regardless of how much IL-15Rc was produced (Fig. 2a, b, c). Simply put, NK-cell activation is achieved once the threshold of IL-15Rc has been reached. Our results are intriguing as IL-15Rc is understood to result in stronger responses and better survival in cancer models. However, it appears that long-term survival depends on which cell type gets activated and we find that different forms of IL-15 activate different cell types.

Our depletion studies demonstrate that in both IL-15 models, CD4^+^ and CD8^+^ T-cells are essential for the immune response. It may at first come as a surprise that NK-cell depletion did not abolish immunity in the IL-15Rc model. However, our data suggests that NK-cells trigger an ‘unfortunate’ immune response. Our studies show that by depleting NK-cells (Fig. 3d) we disrupted this fast-acting innate immune response, giving the T-cells time to launch an adaptive immune response. The NK-depleted IL-15Rc cohort was the only group injected with IL-15Rc-secreting leukemia cells that showed 100% survival. This suggests that taking out NK-cells can be advantageous to the host.

A small number of IL-15Rc-secreting leukemia cells remains and perseveres for many weeks in vivo, suggesting that the lack of target cells cannot be blamed for the absent adaptive immune response and protective immunity. It is therefore possible that these conditions lead to an environment conducive for the development of immune tolerance, resulting in the establishment of a dynamic state of cancer equilibrium.

We also investigated why T-cells in the IL-15Rc model were not able to initiate long-lasting immunity as seen in the IL-15sol model, especially as IL-15Rc-secreting leukemia cells expand rapidly after relapse. T cell exhaustion is associated with cancer and chronic disease [19]. Thus, we examined whether mice showing disease progression in the day 50^+^ IL-15Rc-model exhibited signs of T-cell exhaustion. Programmed cell death protein 1 (PD-1) is the major inhibitory receptor regulating T-cell exhaustion, and T-cells with high PD-1 expression have been demonstrated to lose the ability to eliminate cancer cells [20]. Strikingly, both CD8^+^ and CD4^+^ T-lymphocytes displayed increased PD-1 expression in the IL-15Rc but not the IL-15sol-model (Fig. 6a). In addition, we found CD8^+^ T-cells were significantly more abundant in day 50^+^ IL-15Rc-injected mice (Fig. 6b). IL-15Rc-activated CD8^+^ T-cells further displayed an increase in expression of activation markers CD44, CD25 and GrzB (Fig. 6c-e). This suggests that the continuous long-term exposure to IL-15Rc causes T-cells in the peritoneum of IL-15Rc mice to become exhausted, allowing the cancer cells to proliferate and mice to perish.

In both the initial and later stages of the IL-15Rc-initiated immune response we saw an abundance of NK1.1^+^ cells (Fig. 6f). It is well known that IL-15 induces NK, NKT and ILCs, however, many studies do not specify the form of IL-15 [10, 21, 22]. Conversely, Mortier et al. showed that NK-cells required trans-presentation of IL-15Rc for activation [7]. The value of NK1.1^+^ cells in tumor prognosis has been controversial. Mundy-Bosse et al. found that highly cytotoxic NK-cells were associated with poor prognosis in patients with T-cell lymphoma [23]. In the present study, we also found IL-15Rc-activated NK-1.1^+^ cells to be negatively correlated with disease prognosis and survival.

Waickman et al. reported that IL-15Rc, but not IL-15sol, induced Treg-cell generation [15]. Tregs in the tumor microenvironment are linked to poor prognosis, as they have been thought to suppress tumor immunity, inhibiting the body’s ability to control the growth of cancer cells [24]. We tested whether immune tolerance had developed, which would allow the leukemia cells to proliferate in vivo. While effector cell numbers were similar between the two models, we saw a trend towards higher numbers of T-reg cells in the IL-15Rc-model over IL-15sol (*p* = 0.2054) (data not shown).

In conclusion, the data presented here suggests that the two forms of IL-15 either activate different arms of the immune system (Fig. 7), or that the different arms of the immune system have different requirements when it comes to the presentation of IL-15, explaining the benefit of the two forms of IL-15 in the first place. It is widely accepted that the innate and adaptive arms of the immune system are not two separate entities, but are intimately intertwined to regulate a wide variety of immune responses - maybe with the help of IL-15. Understanding the multifaceted nature of IL-15 remains worthy of further exploration, as comprehending the roles of IL-15sol vs IL-15Rc and the dynamic interplay between them may ultimately provide novel cancer treatments. Furthermore, while IL-15sol may not be the physiologically active form of IL-15 in vivo, our study clearly outlines that there may be advantages of its use in immunotherapies over IL-15Rc.

## Data Availability

All data generated or analysed during this study are included in this published article.

## Abbreviations

FCS: Fetal calf serum
G-CSF: Granulocyte-colony stimulating factor
GFP: Green fluorescent protein
GM-CSF: Granulocyte-macrophage colony-stimulating factor
GrzB: Granzyme B; hr., hour
IFN-γ: Interferon-γ
IL-15Rc: IL-15 receptor complex
IL-15sol: Soluble IL-15
ip: Intra-peritoneal
IP-10: IFN-γ-inducible protein 10
KC: *Chemokine* ligand 1 (CXCL1)
LV: Lentivirus
MCP-1: Monocyte chemoattractant protein-1
MIG: Monokine induced by IFN-γ (CXCL9)
NK-cell: Natural killer cell
ON: Over night
PBS: Phosphate buffered saline
vs: Versus
